# Computational modelling unveils how epiblast remodelling and positioning rely on trophectoderm morphogenesis during mouse implantation

**DOI:** 10.1371/journal.pone.0254763

**Published:** 2021-07-28

**Authors:** Joel Dokmegang, Moi Hoon Yap, Liangxiu Han, Matteo Cavaliere, René Doursat

**Affiliations:** 1 Centre for Advanced Computational Science (CfACS), Manchester Metropolitan University, Manchester, United Kingdom; 2 Complex Systems Institute, Paris Ile-de-France (ISC-PIF), National Centre for Scientific Research (CNRS), Paris, France; 3 NSF-Simons Center for Quantitative Biology, Northwestern University, Evanston, Illinois, United States of America; Laboratoire de Biologie du Développement de Villefranche-sur-Mer, FRANCE

## Abstract

Understanding the processes by which the mammalian embryo implants in the maternal uterus is a long-standing challenge in embryology. New insights into this morphogenetic event could be of great importance in helping, for example, to reduce human infertility. During implantation the blastocyst, composed of epiblast, trophectoderm and primitive endoderm, undergoes significant remodelling from an oval ball to an egg cylinder. A main feature of this transformation is symmetry breaking and reshaping of the epiblast into a “cup”. Based on previous studies, we hypothesise that this event is the result of mechanical constraints originating from the trophectoderm, which is also significantly transformed during this process. In order to investigate this hypothesis we propose MG# (*MechanoGenetic Sharp*), an original computational model of biomechanics able to reproduce key cell shape changes and tissue level behaviours *in silico*. With this model, we simulate epiblast and trophectoderm morphogenesis during implantation. First, our results uphold experimental findings that repulsion at the apical surface of the epiblast is essential to drive lumenogenesis. Then, we provide new theoretical evidence that trophectoderm morphogenesis indeed can dictate the cup shape of the epiblast and fosters its movement towards the uterine tissue. Our results offer novel mechanical insights into mouse peri-implantation and highlight the usefulness of agent-based modelling methods in the study of embryogenesis.

## Introduction

A critical milestone of mammalian development is reached when the embryo implants in the maternal uterine tissue [1, 2]. Prior to implantation, a series of cell fate decisions concomitant with multiple rounds of divisions gradually transform the initial zygote into a blastocyst featuring three different cell lineages: a spherical embryonic epiblast (EPI) wrapped into two extraembryonic tissues, the trophectoderm (TE) and primitive endoderm (PE/VE) [3, 4]. Upon implantation, the embryo moves towards maternal sites, and undergoes significant remodelling, culminating in the case of the mouse in an egg cylinder, a body structure essential to post-implantation phases such as gastrulation [4–6]. A key feature of this blastocyst-to-egg-cylinder transition, still poorly understood, is the appearance of symmetry breaking within the epiblast characterised by its reshaping into a cup [4, 7], which occurs roughly between stages E4.5 and E5.5 of embryonic development.

Many of the main structural changes that occur during implantation have been explained in terms of chemical signals within and between embryonic and extraembryonic compartments [1, 8]. For instance, it was shown that at the onset of implantation epiblast cells exit their naive pluripotency state, self-organise into a highly polarised rosette, and initiate lumenogenesis under the influence of *β*1-integrin signalling [7, 9]. Shortly after implantation, *β*1-integrin enables pro-amniotic cavity formation along the entire egg cylinder via the resolution of multiple rosettes both in extraembryonic cell populations and at their interface with the embryonic tissue [6]. Moreover, differentiation of the primitive trophectoderm into polar and mural trophectoderm leading to the formation of a boundary between the two tissues was traced back to fibroblast growth factors (FGFs) signalling [10].

**Fig 1:**
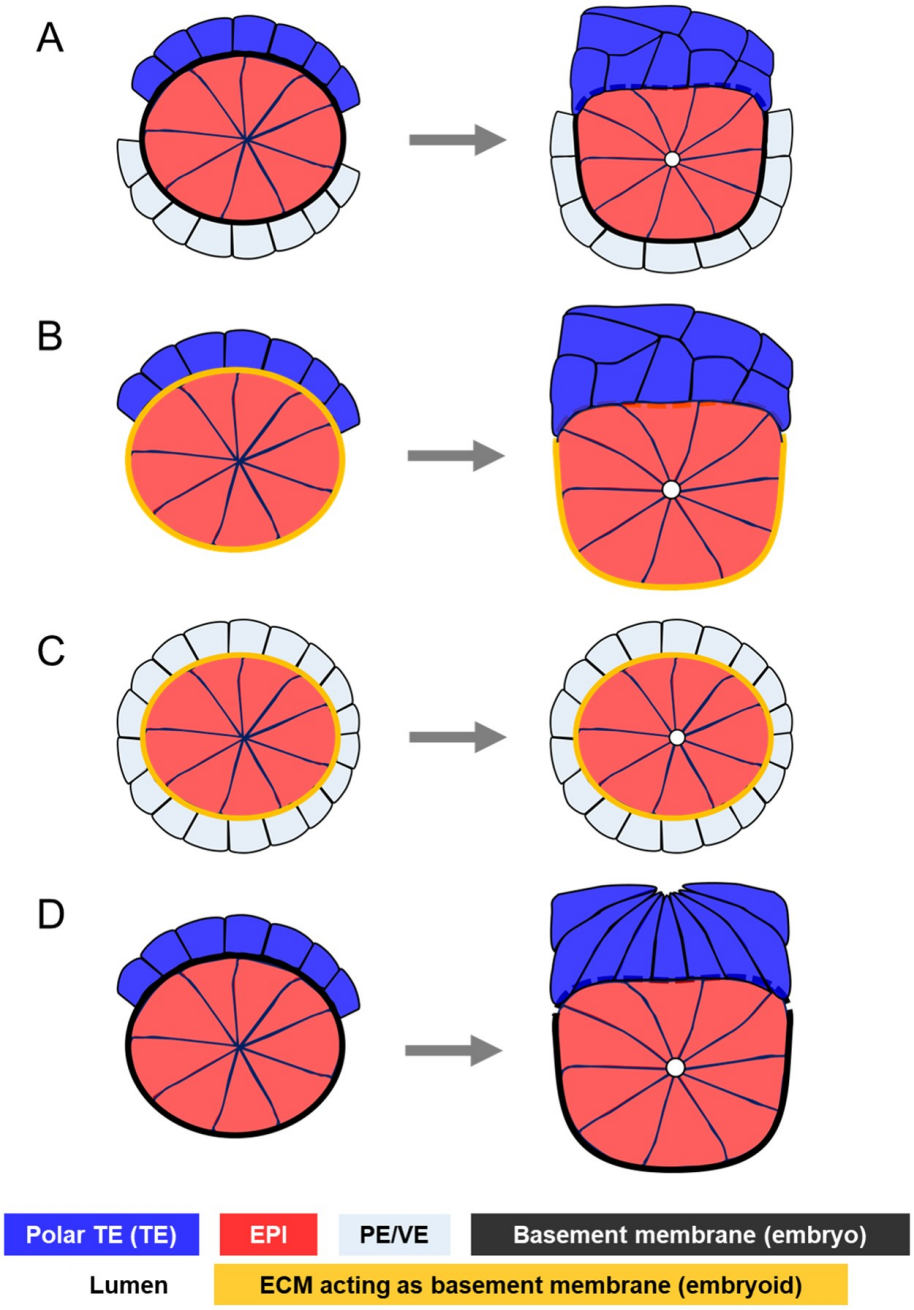
Review of epiblast symmetry breaking theories. **A**. The basement membrane separating the epiblast and the primitive endoderm moulds the epiblast into a cup while it disintegrates between the epiblast and the trophectoderm in mouse embryos [4]. **B**. Embryoid structures featuring epiblast and trophectoderm stem cells surrounded by an ECM acting as a basement membrane (ETS-embryoids) replicate mouse embryogenesis by forming body structures similar to those observed in normal embryonic development [13]. Here the presence of the trophecdoderm shows that this tissue might be required for symmetry breaking in the epiblast and cup shape acquisition. **C**. Embryoid structures featuring epiblast and primitive endoderm stem cells surrounded by an ECM acting as a basement membrane (EXE-embryoids) do not break symmetry in the epiblast, but initiate lumenogenesis [14]. This evidences the requirement of the trophectoderm for the remodelling of the epiblast. **D**. Trophectoderm morphogenesis during mouse implantation. Trophectodermal cells elongate, then undergo apical constriction, resulting in the tissue folding [10]. This suggests that epiblast remodelling into a cup might be a mechanical response to trophectoderm dynamics.

As D’Arcy Thompson already noted about genetics, however, development cannot be construed solely in terms of biochemical signals either: the mechanical interactions between cells and tissues equally and reciprocally contribute to embryogenesis [11, 12]. On the subject of the epiblast remodelling into a cup, a series of biological works have paved the way and triggered further investigation into the mechanics involved. Because it was observed that the EPI did not initiate specific tissue-level symmetry-breaking behaviours, one study stated that after the basement membrane disintegrated between the EPI and TE, the membrane between the EPI and the PE acted like a basket that moulded the epiblast into its cup shape [4] (Fig 1A). Although this hypothesis put the spotlight on the basement membrane, it also suggested that the TE in direct contact with the EPI could play a role in this shape change. Evidence supporting this hypothesis grew when “ETS-embryoids” (ETS: embryonic and trophoblast stem-cell) assembled *in vitro* from EPI and TE stem cells, surrounded by the extracellular matrix (ECM) acting as the basement membrane, replicated embryonic transition from blastocyst to egg cylinder [13] (Fig 1B). Furthermore, a recent study highlighted more clearly the role of the trophectoderm [14]. In this study, ExE-embryoids (ExE: extra-embryonic ectoderm), cultured from EPI and PE stem cells separated by an ECM basement membrane, did not break the symmetry of their initial spherical shape (Fig 1C). In contrast, both ETS- and ETX-embryoids (ETX: embryonic, trophoblast and extra-embryonic endoderm) made from all three blastocyst lineages did reproduce the symmetry breaking observed in real embryos. Together, these studies established the necessity of the trophectoderm for the remodelling of the epiblast [13].

On the other hand, *how* exactly trophectoderm morphogenesis influences shape change in the epiblast has not been elucidated yet because very little is known on trophectoderm morphogenesis during implantation. In the light of recent detailed descriptions of extra-embryonic tissues morphogenesis during implantation [10], it appears increasingly plausible that trophectoderm morphogenesis regulated epiblast remodelling via mechanical interactions at their common boundary. This study showed that polar trophectodermal cells exhibited drastic morphological changes throughout the implantation period. Whereas early implanting blastocysts featured squamous cells in the polar trophectoderm, these cells, driven by a high mitotic and space restrictions due to the formation of a boundary with the mural trophectoderm, later transited to cuboidal, then elongated to acquire columnar shapes. These changes were followed by apical constriction resulting in the folding of the whole tissue, and invagination of the epiblast (Fig 1D). Moreover, this study provided experimental evidence that other structural changes, most notably the stretching of PE cells, resulted from TE morphogenesis [10]. Hence, we want to investigate the hypothesis that trophectoderm morphogenesis drives the remodelling of the epiblast into a cup via mechanical interactions at their common boundary.

Building on the increasing power of computational modelling in developmental biology [15–19], we examine the influence of trophectoderm morphogenesis on the epiblast. The requirement of dramatic cell shape changes in trophectodermal cells, notably apical constriction [10], orients modelling options toward the family of deformable cell models (DCM) [20]. In this category, two classes of models have been predominant in recent research: vertex models (VM) and sub-cellular element models (SEM). Although vertex models were used extensively to study epithelial dynamics [21, 22], discriminatory mechanical behaviours between subsets of cells is not trivial in global energy-based approaches. Hence, we set our choice on SEM, where cells are represented by an agglomeration of computational particles interacting with one another via short-range potentials emulating the viscoelastic properties of their cytoskeleton [23–25]. However, in order to exhibit realistic cell shapes, SEM generally involve a large number of particles, many of which reside within the cell, thus do not have a direct influence on cell shape. This leads to increased computational complexity, limiting the size of cell populations that can be simulated.

Here, we present a novel computational SEM called MG# (*MechanoGenetic Sharp*), which focuses on 3D cell shapes while reducing computational complexity by distinguishing between membrane particles and a single intracellular particle. Using this model, we first uphold the experimental observation that repulsion at the apical surface is sufficient for lumenogenesis in the epiblast. Then, we reproduce trophectoderm morphogenesis during implantation and we provide theoretical support that epiblast remodelling into a cup shape and its movement towards the maternal uterine tissue can be explained by trophectoderm morphogenesis. We also conduct a sensitivity analysis, where we show how different sets of model parameters influence simulation outcomes.

## Model

**Fig 2:**
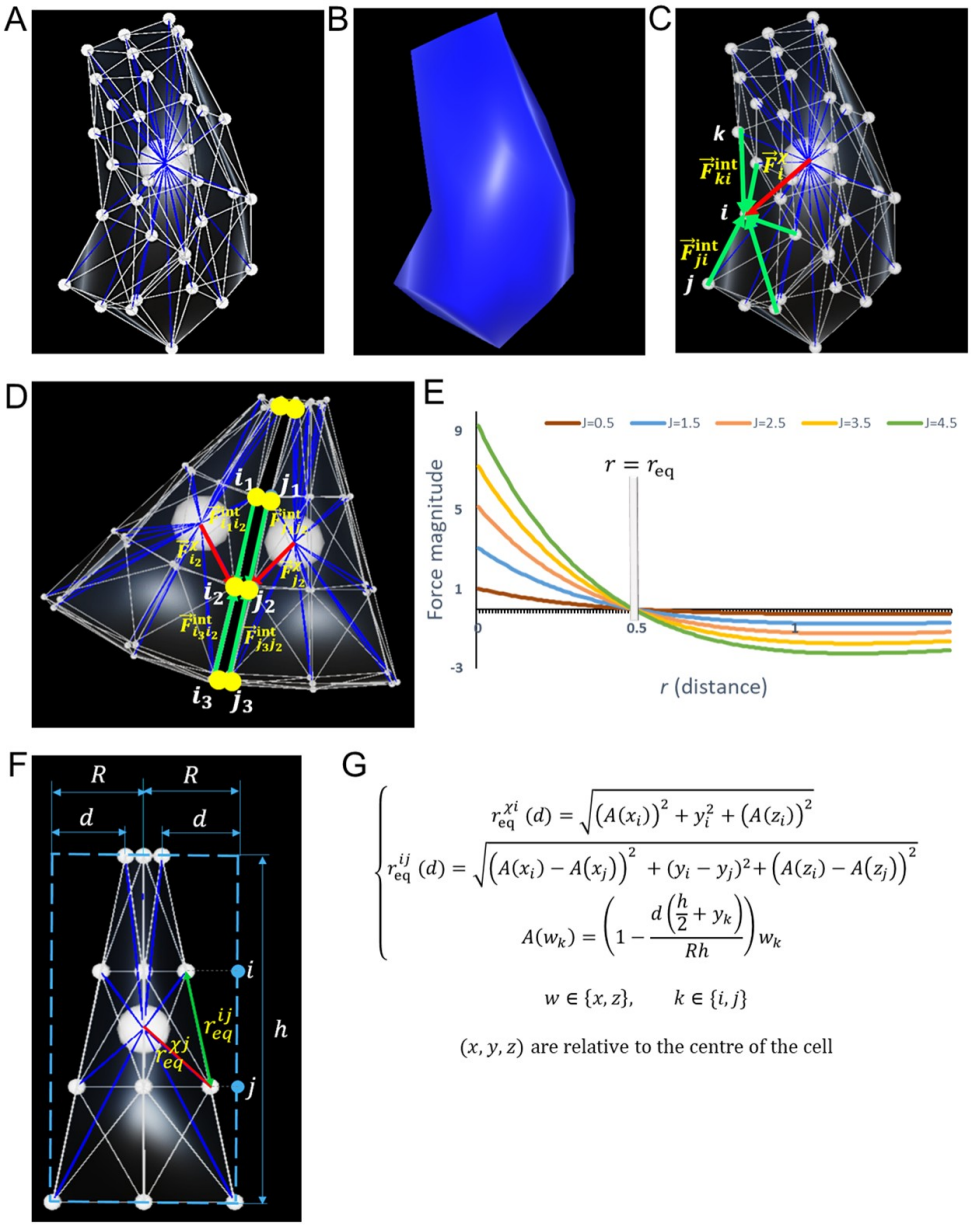
Computational model. **A**. 3D representation of a cell: The cell is abstracted by an agglomeration of particles (small white spheres, 34 in the picture), whose triangulation (white edges) forms the membrane, and by an intracellular particle (big white sphere). Interactions between the intracellular and membrane particles (blue lines) mimic the cytoskeleton. **B**. 3D rendering of a cell without its sub-cellular elements. **C**. Forces acting within a cell: ${\vec{F}}_{ji}^{\text{int}}$, ${\vec{F}}_{ki}^{\text{int}}$ are the forces that membrane particles *j*, *k* exert on another membrane particle *i*. ${\vec{F}}_{i}^{\chi }$ is the force that the intracellular particle *χ* exerts on *i*. **D**. External forces acting on a cell via its particles. Here, ${\vec{F}}_{i_{2}}^{\text{ext}}={\vec{F}}_{j_{2}i_{2}}^{\text{ext}}=\left({\vec{F}}_{j_{1}j_{2}}^{\text{int}}+{\vec{F}}_{j_{3}j_{2}}^{\text{int}}\right)+{\vec{F}}_{j_{2}}^{\chi }$. **E**. Plots of the magnitude of Morse forces under different values of *J*, with *ρ* = 1 and *r*_*eq*_ = 0.5. **F**. Apical constriction of an epithelial cell with original radius *R* shrinking by *d*. **G**. Formulas of the new equilibrium lengths in an apically constricted cell.

Based on the fundamental principles of DCM, our abstraction of the biological cell features particles in interaction under the influence of conservative forces. Emphasis is put on particles at the surface of the cell membrane, bringing our model close to VM [26], while at the same time we also include a single intracellular particle reminiscent of the cell’s micro-tubule organising centre (Fig 2A and 2B).

On the cell membrane, we define a topological neighbourhood based on a triangulation of particles’ positions. Two same cell particles are deemed internal neighbours if they both belong to one of the mesh triangles (Fig 2A). We also define an external neighbourhood based on distances between particles of different cells (Fig 2D). To minimise the computation time required, we introduce cell-cell neighbourhood relationships where particles of different cells are tested for external neighbour links only when the cells to which they belong were already approved as neighbours. Here, a Moore neighbourhood in 3D, well suited for the lattice-like layout of our cells, is favoured. In this setting, a central cell can established neighbourhood relationships with up to 26 neighbours (8 in its plane, 9 in the plane above, and 9 in the plane below).

In order to induce intrinsic mechanical behaviours within cells, we assimilate internal particle neighbourhood links to non-linear springs, which have been shown to faithfully emulate living matter [27]. These springs mimic the activity of actomyosin and microtubule networks in the cytoskeleton, and forces are derived from their elastic potential (Fig 2C–2E). In the cell’s resting state, the equilibrium distance of each spring coincides with the length of the segment formed by its nodes. Cell dynamics arise from alterations to these equilibrium distances. In apical constriction for instance, new equilibrium lengths are computed as in Fig 2F and 2G.

### Equation of motion

Acting on a given membrane particle *i*, we distinguish four main types of forces: internal forces ${\vec{F}}_{ji}^{\text{int}}$ which act on the cell membrane, mimmicking line and surface tensions, cytoskeleton forces ${\vec{F}}_{i}^{\chi }$, external forces ${\vec{F}}_{ji}^{\text{ext}}$, and specific forces ${\vec{F}}_{i}^{\text{spe}}$, which are optional and can be restricted to a specific phenomena, for instance, repulsives forces at the origin of lumen creation. Biological media are often characterised by a low Reynolds number, due to their high viscosity, which minimises the effects of inertia [17, 19–21]. We therefore subject particles to an over-damped, first-order equation of motion:

$$\begin{array}{c} (\sum_{j\in N_{\text{int}}\left(i\right)}{\vec{F}}_{ji}^{\text{int}})+{\vec{F}}_{i}^{\chi }+(\sum_{j\in N_{\text{ext}}\left(i\right)}{\vec{F}}_{ji}^{\text{ext}})=\lambda_{\text{med}}{\vec{v}}_{i} \end{array}$$ (1)

where $N_{\text{int}}\left(i\right)$ and $N_{\text{ext}}\left(i\right)$ represent the sets of internal and external neighbours of particle *i*, and λ_med_ is the coefficient of friction exerted on all membrane particles.

In line with Newton’s third law of motion, membrane particles entertain reciprocal forces equal in magnitude and opposite in direction with the intracellular particle. Therefore, the dynamics of the nucleus is dictated by:

$$\begin{array}{c} \sum_{i}-{\vec{F}}_{i}^{\chi }=\lambda_{\chi }{\vec{v}}_{\chi } \end{array}$$ (2)

where λ_χ_ is the coefficient of friction exerted on the intracellular particle.

### Internal and cytoskeleton forces

The internal force created by a particle *j* on a neighbouring particle *i* derives from a Morse potential (Fig 2E). Previous studies have used Morse potentials to represent forces in a biological context [23, 25]. The expression of this force is given by:

$$\begin{array}{c} {\vec{F}}_{ji}^{\text{int}}=2J_{\omega }\rho \;\left(e^{2\rho (r^{ij}-r_{\text{eq}}^{ij})}-e^{\rho (r^{ij}-r_{\text{eq}}^{ij})}\right)\;{\vec{u}}_{ij} \end{array}$$ (3)

where *J*_*ω*_ represents the interaction strength between particles *i* and *j*, both of cell type *ω* (*ω*∈ {TE, EPI}), *r*^*ij*^ is the distance between *i* and *j*, $r_{\text{eq}}^{ij}$ is the equilibrium-length of the spring force between *i* and *j*, and ${\vec{u}}_{ij}$ is the unit vector along the direction formed by *i* and *j*. Similar forces dictate interactions between the intracellular particle and the membrane particles.

### External forces

Given the tight packing in epithelial tissues, a cell membrane is always in contact with neighbouring cell membranes. Thus local action on a membrane produces an equivalent deformation on the surrounding cells. In other words, a particle always transmits the force received to its external neighbours. To account for this behaviour, we submit particles and their external neighbours to equal forces. This is done by setting the external force acting on a particle to be equal to the sum over all its external neighbours of their internal and nucleus forces:

$$\begin{array}{c} {\vec{F}}_{i}^{\text{ext}}=\sum_{j\in N_{\text{ext}}\left(i\right)}{\vec{F}}_{ji}^{\text{ext}} \end{array}$$ (4)

$$\begin{array}{c} {\vec{F}}_{ji}^{\text{ext}}=(\sum_{k\in N_{\text{int}}\left(j\right)}{\vec{F}}_{kj}^{\text{int}})+{\vec{F}}_{j}^{\chi } \end{array}$$ (5)

Model parameters are summarised in Table 1.

**Table 1:** Model parameters.

Name	Description
*J*_TE_	Particles interaction strength for trophectoderm cells
*J*_EPI_	Particles interaction strength for epiblast cells
*ρ*	Morse scaling factor
λ_med_	Friction coefficient for viscous forces in the biological medium
λ_χ_	Friction coefficient for viscous forces within the cell
$r_{\text{eq}}^{ij}$	Distance between particles *i* and *j*
$r_{\text{eq}}^{ij}$	Equilibrium distance between particles *i* and *j*
*R*	Apical radius of a cell
*h*	Cell height
*d*	Shrinkage rate of apical radius during apical constriction

We implemented this model in C# into an open source modelling platform that we named MG# (standing for *MechanoGenetic Sharp*). This tool features a simulation engine and a 3D viewer. The source code for the simulation engine can be found at https://github.com/guijoe/MGSharpCore, while the repository for the Unity3D-based viewer can be found at https://github.com/guijoe/MGSharpViewer. Each Simulation of mouse implantation with MG# as described in the following section required about 4 minutes in average on an laptop powered with an *Intel(R) Core(TM) i7–6600* CPU and 16GB of RAM.

## Results

In this section, we applied our model to the study of mouse embryo morphogenesis during implantation. Here we focused on epiblast and trophectoderm tissues. First, we tested the hypothesis of whether repulsion at the apical surface of the epiblast was sufficient to account for lumenogenesis. Then, we simulated both tissues’ morphogenesis and showed that the epiblast remodelling into a cup shape and its movement towards the maternal uterine tissue could be explained by trophectoderm morphogenesis. Next, we conducted a sensitivity analysis, to show how different sets of parameters influenced simulation outcomes.

### Repulsion at the apical surface of the epiblast facilitates lumenogenesis

The study of how lumens arise in epithelial tissues has revealed two predominant mechanisms: cavitation mediated by apoptosis, and hollowing, in which the lumen is formed by exocytosis and membrane separation [28, 29]. In the case of highly polarised epithelia, it was shown that cavitation was not necessary for lumenogenesis [30]. Hence, the hollowing mechanism was privileged in epiblast lumenogenesis, which features highly polarised cells spatially organised in the shape of a rosette. Moreover, it was hypothesised that repulsion mediated by anti-adhesive molecules such as podocalyxin (Podxl) drove lumen formation in the epiblast [4, 7, 9, 14]. Furthermore, evidence for hollowing in the epiblast was observed in a recent study [14], where apoptosis was found not to regulate lumenogenesis, but *Podxl* was discovered to be predominant at the apical surface of cells facing the lumen.

**Fig 3:**
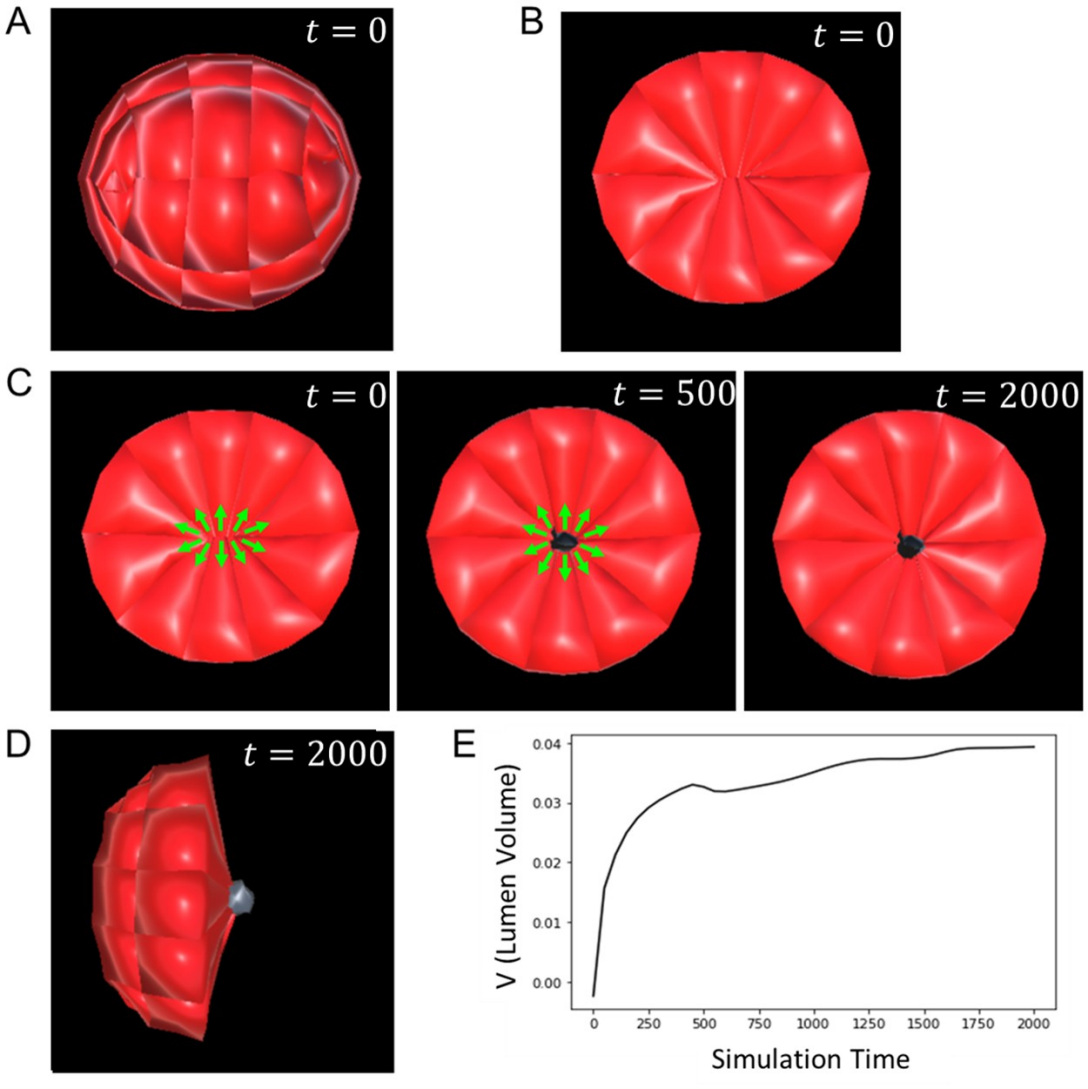
Lumenogenesis in the epiblast. **A**. A 3D model of a rosette-shaped epiblast. **B**. A 2D slice of the epiblast in **A** showing apically constricted cells of the building block of the epiblast rosette. **C**. Creation of the lumen cavity by repulsion at the apical surface of the epiblast. Green arrows represent the direction of repulsive forces. The snapshots (from left to right) were taken respectively at *t* = 0, 500 and 2000. **D**. Lateral view of the sliced epiblast showing the lumen volume. The lumen has been greyed to allow a better view over the black background. **E**. Evolution over time of the volume of the lumen. Values of the equation parameters: *J*_EPI_ = 2.5, λ = 2, *ρ* = 1, *R*_lum_ = 0.25.

Using our model, we sought to determine theoretically whether hollowing via repulsion at the apical surface of the epiblast rosette was a viable mechanism for lumenogenesis in this tissue. First, we built a 3D rosette-shaped epiblast by submitting polarised epithelial cells to apical constriction [7] (Fig 3A and 3B, S1A Fig). Then, inspired by the anti-adhesive role of *Podxl*, we broke adhesive links between cell membranes in contact at the apical surface of the rosette, meaning that certain neighbouring pairs of particles were not more submitted to the exact same forces, but rather could be repelled in different directions.

Though not the only factors regulating lumen creation, it has been shown that repulsive forces driven by electrical charges facilitate hollowing in the epiblast [7, 31, 32]. For simplification purposes, we created a virtual source (*O*) at the centre of the rosette to exert repulsive forces on apical particles. To model these effects, we used conservative forces from a Morse potential (Eq 6).

$$\begin{array}{c} {\vec{F}}_{i}^{\text{rep}}=2J_{\text{EPI}}\rho \;\left(e^{2\rho (r^{Oi}-R_{\text{lum}})}-e^{\rho (r^{Oi}-R_{\text{lum}})}\right)\;{\vec{u}}_{Oi} \end{array}$$ (6)

where *R*_lum_ is the radius of the lumen.

These forces prompted neighbouring apical particles and surfaces to drift apart from each other, initiate the creation of a lumen at the centre of the rosette (Fig 3C–3E). This result, upholding experimental data, suggests that hollowing via loss of adhesion and apical repulsion are necessary for lumenogenesis in the mouse epiblast.

### Mechanical constraints imposed by TE morphogenesis on the epiblast drive cup shape acquisition

A key feature of the blastocyst-to-egg-cylinder transition is the symmetry breaking within the epiblast and its shaping into a cup [4, 7]. During this transformation, the epiblast remodels from an oval ball to a tissue with a flat surface at its boundary with the trophectoderm. Previous studies have established the requirement of the trophectoderm in this shape change [13, 33]. Using the presented model, we investigated how trophectoderm morphogenesis influenced the cup shape acquisition by the epiblast. Our simulation protocol consisted of reproducing the sequence of morphological events observed in the trophectoderm as described in [10] (elongation followed invagination via apical constriction), and keeping track of the consequent changes in the epiblast. For simplicity and to keep the model computationally efficient, we assumed that there were no cell divisions in the tissue.

**Fig 4:**
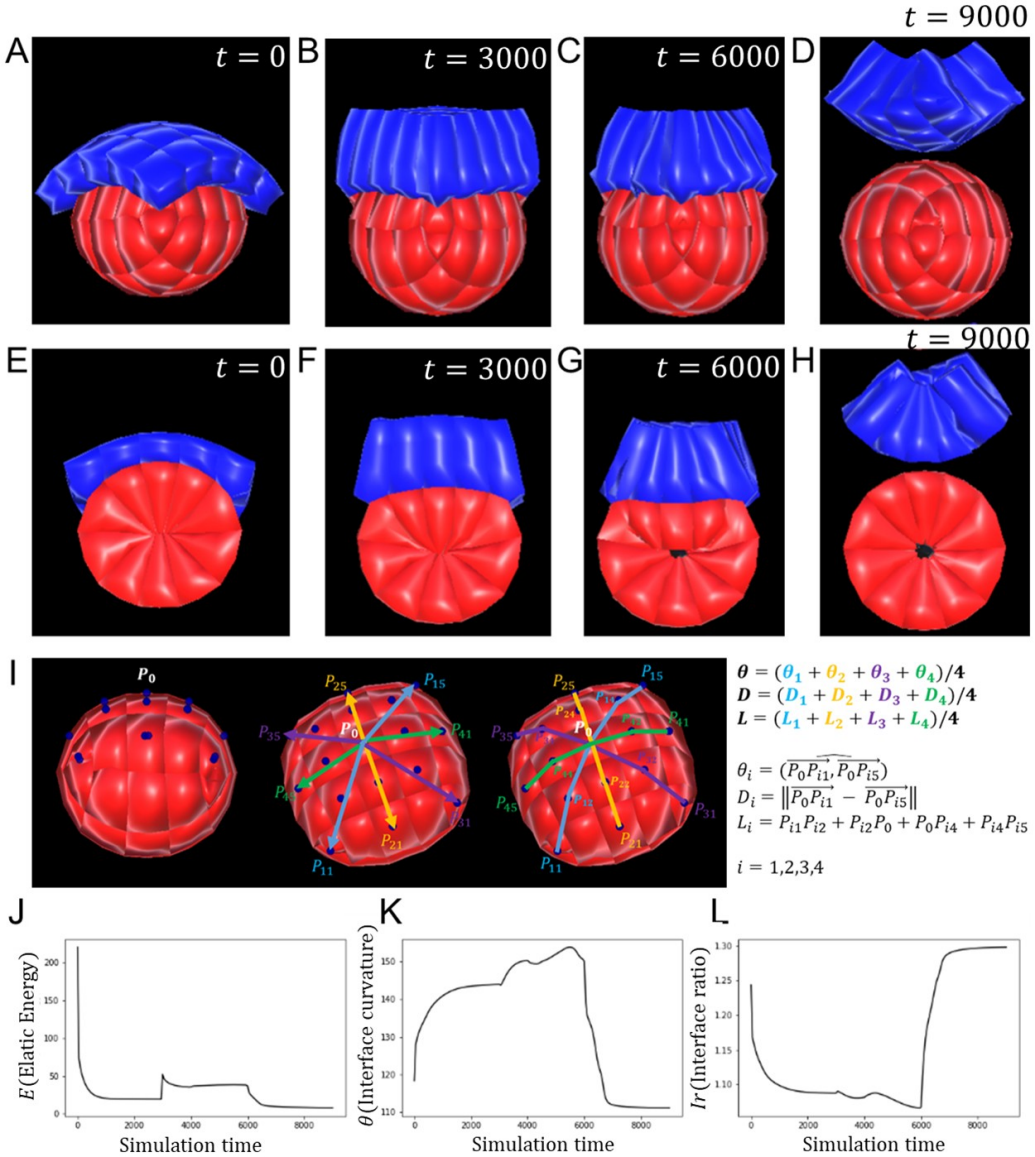
Trophectoderm morphogenesis regulates epiblast shape. **A-D**. 3D snapshots of the simulation of TE and EPI morphogenesis during mouse implantation, and the regulation of EPI shape, taken respectively at *t* = 0, 3000, 6000 and 9000. **E-H**. Corresponding 2D slices of the cell population at the same stages. **(A,E)**. The initial stage features a single layered TE with cuboidal cells resting upon the rosette-shaped epiblast. **(B,F)**. TE cells have transited to a columnar shape. **(C,G)**. The TE has folded by apical constriction of single cells. Concomitantly, lumenogenesis was initiated in the epiblast (the process starts at *t* = 4000). **(D,H)**. After adhesive links were broken between TE and EPI, the EPI bounces back to its near spherical shape. **I**. Definitions of the metrics used to evaluate the model, involving the curvature *θ*, TE/EPI interface diameter *D*, TE/EPI interface length *L*, and interface ratio *L*/*D*. **J**. Plot of the population’s elastic energy *E*. Discontinuities mark the start of new morphological events at *t* = 0, 3000, 4000, and 6000). After removal of the TE, *E* falls closer to zero than ever before, meaning that cells are closer to their resting stage, hence less externally constrained. **K**. Plot of the interface curvature *θ*. During TE morphogenesis, *θ* rises towards a flat angle, then sharply drops when the TE is removed. **L**. Plot of the interface ratio *L*/*D*. During TE morphogenesis, the interface curvature decreases towards 1, then sharply increases when the TE is removed. Values of the equation parameters: *J*_EPI_ = *J*_TE_ = 2.5, λ_med_ = λ_χ_ = 2, *ρ* = 1, *d* = 0.5.

We built a virtual embryo consisting of a TE sheet with initial cuboidal cells laying on top of an oval rosette-shaped epiblast (S1B Fig). At the initial stage (Fig 4A and 4E), new equilibrium lengths were computed for all TE cells, with the goal of triggering a transition from cuboidal cells to more elongated columnar shapes with smaller apical surface. These cells lost their resting state and regained it by gradually aligning their actual springs lengths with the calculated equilibrium lengths (Fig 4B and 4F). After that, we initiated invagination in the TE. Single cell mechanisms at work are often activated in discriminatory ways both in space and time [34–36]. In our simulations, the distribution over the entire sheet of the length *d* by which the apical radius of cells *R* was shrunk depended on the position of the cell in relation to the centre of the sheet via a step function: cells in the middle of the sheet were set to constrict completely (*d* = *R*), while cells on the boundary did not constrict (*d* = 0, S2 Fig). The coordinated movement of cells induced by these positional laws caused the tissue to fold and invaginate the epiblast. Short after TE invagination begins, we initiated lumenogenesis in the epiblast (Fig 4G). In order to highlight the requirement of the TE, following TE invagination (Fig 4C and 4G), we broke the contacts between the TE and the epiblast for the remaining time of the simulation, inhibiting any mechanical interactions between the two tissues, but maintaining both tissues’ own mechanics (Fig 4D and 4H). We noted that throughout the experiment, with the exception of lumenogenesis, epiblast cells did not initiate any behaviours, the epiblast as a whole simply reacted to the mechanics induced by either the presence or the absence of the TE.

To appreciate the impact of the TE on the epiblast, we defined the elastic energy *E*_*i*_ of a cell *i* as the sum over all cell springs of the squared difference between equilibrium and actual lengths. We extended this notion by defining the total elastic energy of a tissue or an entire population of cells as the sum of *E*_*i*_’s in the population (Eq 7).

$$\begin{array}{c} E=\sum_{k\leq N}(\sum_{s\leq N_{k}}{\left(r_{\text{eq}}^{ks}-r^{ks}\right)}^{2}) \end{array}$$ (7)

where *N* is the number of cells in the population and *N*_*k*_ the number of springs in cell k.

Cells always tended to minimise this energy, which can also be viewed as the degree of relaxation of cell: the closer it is to zero, the closer the cell is in its resting state, the more relaxed it is, hence the less constrained. In addition, we monitored the curvature of the epiblast, i.e. the inclination angle *θ* of the epiblast surface covered by the trophectoderm (Fig 4I). An increasing curvature, trending towards a flat surface, was characteristic of the epiblast’s transition from an oval rosette to a cup. Some fluctuations could however be observed at the onset of lumenogenesis in our simulations (Fig 4I). Moreover, we measured the length (*L*) and diameter (*D*) of the interface between EPI and TE, and considered their interface ratio (*L*/*D*) as our third evaluation metric (Fig 4I). It was expected that this ratio would decrease towards 1, and that the curvature would increase towards 180 as a result of the flattening of the epiblast, as observed in [33]. We plotted the profiles of the curvature, the interface ratio and the elastic energy throughout our simulation.

Our model matched biological expectations by replicating, on the one hand, an increasing curvature and a decreasing interface ratio, with ultimately a flat TE/EPI interface just before we removed the TE (Fig 4C, 4G, 4K and 4L; also S1 Video). On the other hand, as soon as the TE was removed, the epiblast bounced back to its original shape (Fig 4D, 4H, 4K and 4L; also S2 Video). This result agrees with the experimental observation that without the TE, the epiblast does not break symmetry [14]. The elastic energy profiles tie these behaviours to the mechanical influence of the TE over the epiblast. Actually, breaking mechanical interactions between the TE and the EPI not only resulted in a sharp drop in elastic energy, but this energy also plateaued at a value significantly lower than in other stages (Fig 4J), demonstrating that cells were more mechanically constrained when both tissues were in contact.

These observations suggest that the presence of the TE imposes mechanical stress on epiblast cells, hinting to the necessity of this tissue’s morphogenesis in the remodelling of the epiblast.

### Trophectoderm morphogenesis fosters epiblast movement towards the uterine tissue

An important requirement of implantation is close contact between the embryo and the uterine tissue. As soon as the three pre-implantation lineages are specified, the blastocyst hatches out of the zona pellucida and initiates the process of implantation [4]. However, there exists a gap between the hatched blastocyst and attachment sites in the uterus. In order to close this gap, movement of the epiblast towards the abembryonic pole is required. It was recently established that this movement of the embryo towards maternal sites occur concomitantly to the drastic morphological changes observed in the TE [10]. Furthermore, it was observed in that same study that primitive endoderm expansion over the whole embryo is driven by TE morphogenesis. Given that the trophectoderm keeps close contact with the epiblast during these events, we hypothesised that epiblast positioning could also be affected by TE morphogenesis. We employed computational modelling to examine whether TE morphological changes could influence the trajectory of the epiblast.

**Fig 5:**
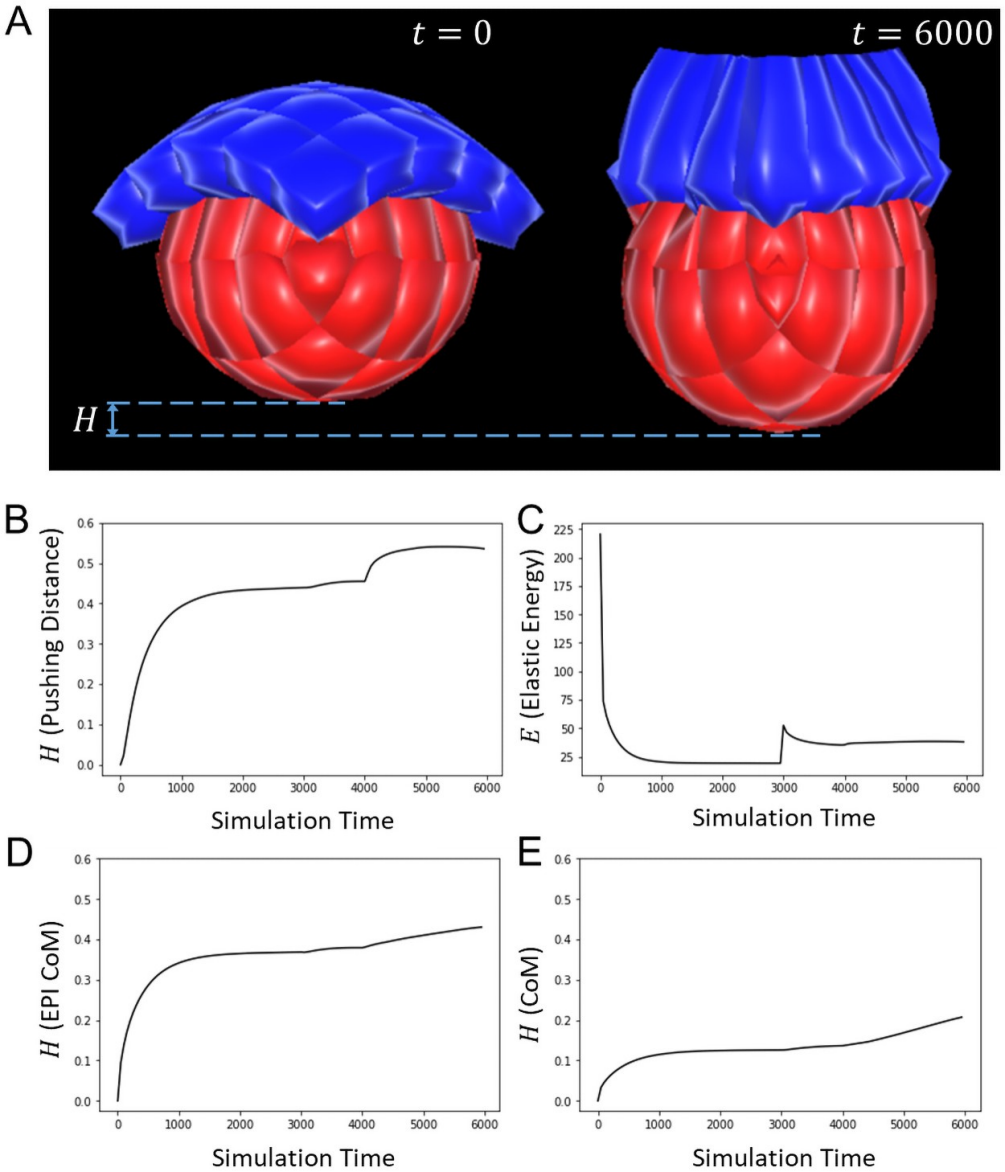
Trophectoderm fosters epiblast movement towards maternal sites. **A**. Snapshots of the simulation of TE and EPI morphogenesis during mouse implantation, and their influence on EPI positioning, taken respectively at *t* = 0 and 6000. **B**. Plot of the pushing distance, which increases with time. **C**. Plot of the elastic energy *E*. Discontinuities mark the start of new morphological events (*t* = 0 and 3000). The sudden soar observed at *t* = 4000 reflects the slight elongation of the tissue due to hollowing-driven lumenogenesis in the epiblast. **D**. Plot of the pushing distance on the epiblast Centre of Mass (CoM), which also increases with time. **E**. Plot of the pushing distance on the cell population Centre of Mass (CoM), which also increases with time. Values of the equation parameters: *J*_EPI_ = *J*_TE_ = 2.5, λ_med_ = λ_χ_ = 2, *ρ* = 1, *d* = 0.5, *R*_lum_ = 0.25.

Here, as previously, we reproduced the sequence of TE morphogenesis (elongation followed by invagination via apical constriction), and observed how it affected the position of the epiblast (which also undergoes lumenogenesis). To highlight how the TE influences the trajectory of the epiblast, we defined what we designated as the “pushing distance”. We computed this distance at any given time point of the simulation by calculating the difference in height between the lowest point of the epiblast at that time point and the lowest point at the initial stage (Fig 5A). We plotted the profiles of this metric and observed an increasing pushing distance as the TE transited from cuboidal to columnar, then as the TE folded (Fig 5B). The sudden soar observed at *t* = 4000 reflects the slight elongation of the tissue due to hollowing-driven lumenogenesis in the epiblast.

We chose to monitor the lower end of the epiblast because it is via this pole that the embryo attaches to maternal sties. However, to ensure that the observed changes did not merely represent an elongation of the epiblast, we also tracked the trajectory of the Centre of Mass (CoM) of both the epiblast (Fig 5D) and the entire cell population (Fig 5E). Similarly, these metrics reaffirmed that the epiblast indeed engages in a downwards movement. Furthermore, we checked that lumenogenesis in the epiblast was not necessary to foster this motion (S3 Fig). These results suggest that TE morphogenesis, while reshaping the epiblast, also fosters the embryo’s movement towards maternal sites.

### Sensitivity analysis

Physical properties are generally a segregating factor between differentiated cells in development [37, 38]. Although the mouse trophectoderm and epiblast form distinct cell lineages and exhibit different properties [39], we have so far assumed similar characteristics for both types of cells. The nature of our model allows for global physical properties such as mechanical stiffness to emerge from lower scale interactions between subcellular elements. In order to characterise cells by their stiffness and thus differentiate trophectoderm and epiblast cells, we first needed to establish how this property depended on intrinsic model parameters.

Parameters related to the dimensions of the simulated epithelial cells in their columnar state were assumed to be non dimensional, hence only represented a ratio. The apical aspect ratio used in our simulations was approximated from measurements in [10] (apical ratio = *height*/*width* ≈ 2, hence *h* = 2 and *R* = 0.5). Furthermore, we assumed that apical constriction tended to reduce cells apical surface to 0, hence found it appropriate to use *d* = 0.5 in all simulations given that *R*, the apical radius is equal to 0.5. Because model parameters values such as particle interaction strengths (*J*) and friction coefficients (λ_med_, λ_χ_) were not based on experimental measures, we set out to to conduct a sensitivity study on this parameters in order to determine how they related to cell stiffness. Other parameters such as distances between particles proceeded from the number of vertices and triangulation used for the meshes of the simulated cells, and were variable between pairs of particles. Here, we also study how they influence simulated biomechanical properties of cells.

**Fig 6:**
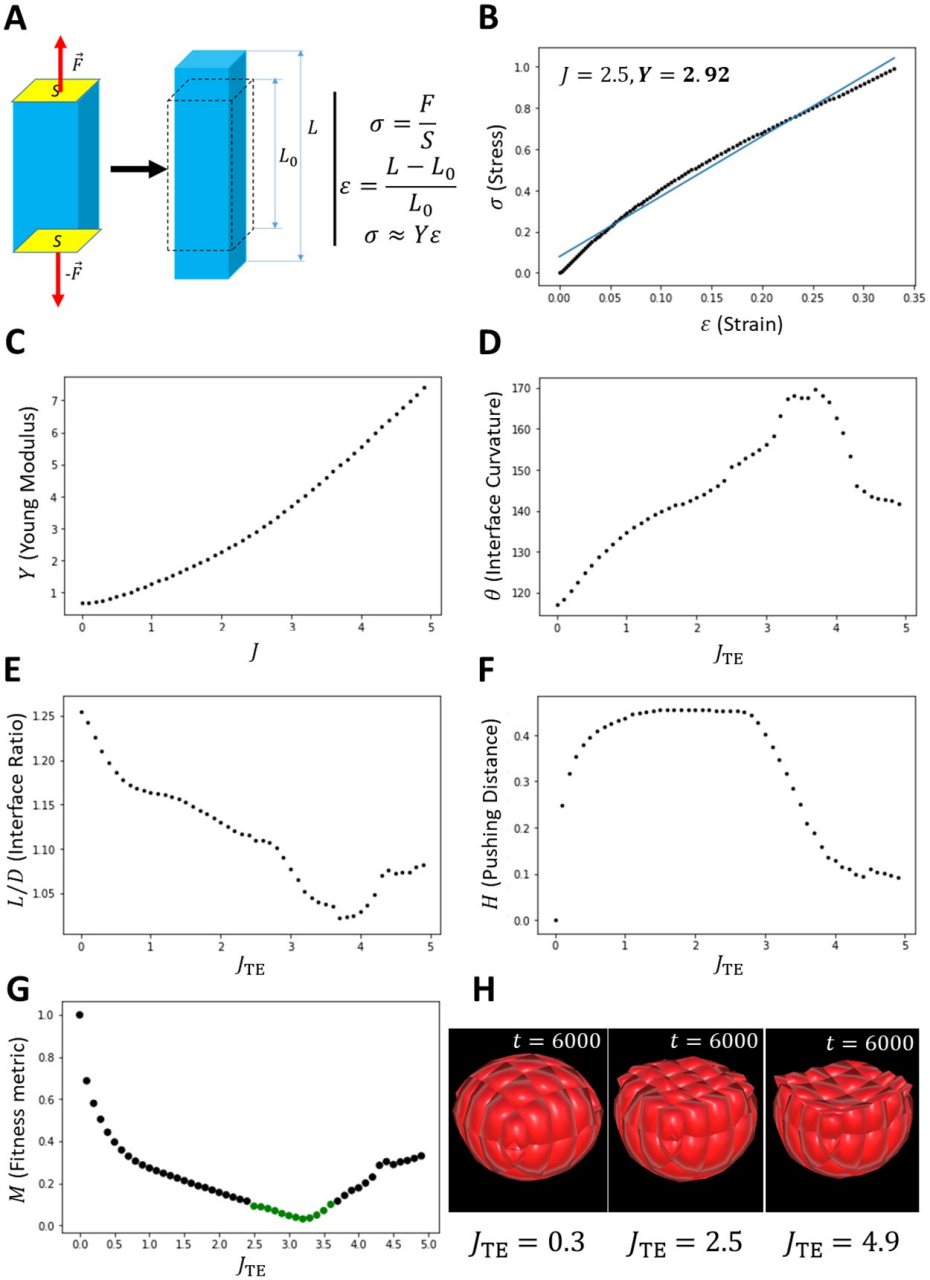
Mechanical properties of EPI and TE determine mouse implantation. **A**. In“Silico” experimental protocol used to determine cells elastic modulus. **B**. Stress-Strain curve (*black*) for a single epithelial cell (34 vertices) with *J* = 2.5. (*blue*) Linear approximation of the Stress-Strain curve. The elastic modulus of the cell is determined by the slope of this line (*Y* = 2.92, *φ* = 2.92*ϵ* + 0.08, *R*_*value*_ = 0.99). **C**. Plot of the Elastic (Young) modulus of cells as a function of parameter *J*, the interaction strength between subcellular particles. **D,E,F**. Respective Plots of the Interface curvature, the Interface ratio and the Pushing Distance as functions of the mechanical stiffness of TE cells (determined by *J*_TE_ as in **C**). **G**. Plot of the fitness metric as functions of the mechanical stiffness of TE cells (determined by *J*_TE_ as in **C**). **H**. Snapshots of the epiblast shape at the end of simulations for different values of *J*_TE_. With equal stiffness (middle, *J*_TE_ = 2.5, *J*_EPI_ = 2.5), trophectoderm morphogenesis flatten the epiblast, which acquires a cup shape. However, with significantly lower stiffness (left, *J*_TE_ = 0.3, *J*_EPI_ = 2.5), trophectoderm morphogenesis barely reshape the epiblast; meanwhile, with considerably higher stiffness (right, *J*_TE_ = 4.9, *J*_EPI_ = 2.5), the trophectoderm invaginates the epiblast, forcing a concave interface with the epiblast. Other parameters values, λ_med_ = λ_χ_ = 2, *ρ* = 1, *d* = 0.5.

We used an “in Silico” adaptation of the experimental protocol described in [40] to estimate cells stiffness based on the computation of a measure of their elasticity modulus (also known as *Young* modulus). For a given value of *J*, we perform a series of simulations consisting of applying forces of increasing magnitudes (*F*) on the apical and basal faces of an epithelial cell (Fig 6A). For each force, we calculate the associated stress ($\sigma =\frac{F}{S}$, where *S* is the surface area of each face) and note the resulting deformation (strain, $\epsilon =\frac{\Delta L}{L_{0}}$). We then plotted the stress-strain curve and estimated the Young modulus (*Y*) as the slope of the curve using a linear regression model (*J* = 2.5 → *Y* = 2.92, Fig 6B). Using this protocol, we ran simulations with 50 different values of *J* uniformly distributed between 0 and 5, recording estimated values of *Y* after every simulation. The plot in (Fig 6C) suggests that *Y* relates to *J* in a measurable way. More broadly, *Y* increases with *J*. In other words, the interaction strength between subcellular particles regulates cells global stiffness: the stronger this interaction is, the stiffer the cell.

We conducted the same analysis on how friction forces coefficients λ_χ_ and λ_med_ affect cell mechanical properties. We fixed λ_med_ (λ_med_ = 2), varied λ_χ_, and observed the evolution of cells elasticity modulus as a function of $\frac{\lambda_{\chi }}{\lambda_{\text{med}}}$. Simulated show that above a certain threshold ($\frac{\lambda_{\chi }}{\lambda_{\text{med}}}\geq 0.161$), cells elasticity modulus was constant (S4A and S4B Fig). Below this threshold, the structure of the cell was compromised (S4C Fig). Overall, these observations suggested that cell mechanical properties did not depend on differences between friction parameters within and without the cell. Furthermore, we refined the cell mesh, taking the number of vertices to 42 (S4D Fig), and repeated the experiments, varying values of parameter *J* (S4E and S4F Fig). Results show that mechanical properties changed with mesh refinement (For *J*_42_ = 2.5 → *Y*_42_ = 2.75). However, while refining the mesh, parameters can be tuned in order maintain cell stiffness (*J*_42_ = 2.6 → *Y*_42_ = 2.90) to allow similar responses to external stress.

Having established how model parameters regulate cells stiffness, we were able to discriminate between cell types based on parameter values we set for each. We then sought to investigate how differences between physical properties of trophectoderm and epiblast cells would influence mouse implantation. For this, we conducted a parameter space exploration in the one dimensional space of values of parameter *J*_TE_, maintaining the value of *J*_EPI_ constant to a value of 2.5. This series of experiments consisted of running 50 different simulations of mouse implantation, with values of *J*_TE_ ranging from 0 to 5 with a step of 0.1. To better appreciate the impact of the trophectoderm on the epiblast, we do not trigger lumenogenesis in the epiblast. For every simulation, we recorded the curvature, interface ratio and pushing distance as defined in previous section, and plotted their values against values of *J*_TE_ (Fig 6D–6F). In order to determine which values of *J*_TE_ perform best overall for these metrics, we defined a normalised fitness measure consisting of a combination of these metrics as previously done in [17]. If we denote by *θ*(*J*_TE_), *Ir*(*J*_TE_) and *H*(*J*_TE_) the respective values of the curvature, interface ratio and pushing distance for a given value of *J*_TE_, and *θ*_min,max_, *Ir*_min,max_ *H*_min,max_ their optimal values in the simulated data, the fitness metric (*M*) is defined by Eq 8.

$$\begin{array}{c} M\left(J_{\text{TE}}\right)=\frac{1}{3}({\left(\frac{\theta \left(J_{\text{TE}}\right)-\theta_{\text{max}}}{\theta_{\text{max}}-\theta_{\text{min}}}\right)}^{2}+{\left(\frac{Ir\left(J_{\text{TE}}\right)-{Ir}_{\text{min}}}{{Ir}_{\text{max}}-{Ir}_{\text{min}}}\right)}^{2}+{\left(\frac{H\left(J_{\text{TE}}\right)-H_{\text{max}}}{H_{\text{max}}-H_{\text{min}}}\right)}^{2}) \end{array}$$ (8)

It can be observed that function *M* admits a minimum and its values are constrained in [0, 1]. We plotted this metric against values of *J*_TE_ and considered that areas where the fitness fell below 0.1 represented simulations featuring a good compromise between curvature, interface ratio and pushing distance (Fig 6G, *green points*). The plotted data hint the existence of a preferential range of values that yield optimal fitness with respect to the three metrics involved (Fig 6G *green points*, Fig 6H *middle*). Within this range, the strength of subcellular interactions is always always higher for trophectoderm cells (*J*_TE_ ∈ [2.5, 3.5]) than for epiblast cells (*J*_TE_ = 2.5). Assuming that cells stiffness remain constant through implantation, this result suggest that mouse implantation requires trophectoderm cells to be generally stiffer than epiblast cells, in agreement with measurements reported in [39]. However, outside of this range, simulations appear to perform poorly. For instance, below this range i.e. with TE cells more ductile than EPI cells, the epiblast is not sufficiently remodelled into a cup (Fig 6H, *left*), as attested by moderate performances of the interface curvature and ratio (Fig 6D and 6E)). Above this range i.e. simulations featuring TE cells significantly more rigid than EPI cells, the trophectoderm considerably invaginates the epiblast, creating a concave interface ((Fig 6H, *right*)). This reflects poorly on the pushing distance as highlighted by the negative slope of its curve (Fig 6F)).

## Discussion and conclusion

Understanding the processes by which the mammalian embryo implants in the maternal uterus is crucial to many breakthroughs in embryology [1]. New insights into these morphogenesis events could be of great importance in helping for example to reduce human infertility [41]. Although advances have been made by studying biochemical cues involved in these events, we focused here on the mechanical basis at the cellular level of epiblast morphogenesis. In order to study the physical dynamics of mouse implantation, we have designed a novel, computationally efficient model of biological cells and tissue mechanics able to simulate key episodes of vertebrate morphogenesis. With this model, we were able to schematically reproduce lumenogenesis in the epiblast, trophectoderm morphogenesis driven by single cells elongation and apical constriction, as well as provide theoretical support to the fact that this morphogenesis regulates the remodelling and positioning of the epiblast during implantation.

The necessity of a model featuring deformable cells arose from the need to simulate drastic cell shape changes involved in mouse embryonic implantation. Our model’s assumption of equal physical forces on particles sharing neighbourhoods in dense epithelial settings essentially brings it close to vertex models. Nevertheless, inspired by SEM approaches, our model also makes use on an intracellular element, explicitly defines pairwise forces between particles, models epithelial cells lateral faces, and exhibits distinct particles at cellular junctions, each belonging to exactly one of the cells involved. With these hybrid properties, MG# metaphorically bridges the gap between the two frameworks. The gains of this approach include more bio-realistic cell shapes, and the relative ease of modelling cell adhesion in epithelial networks, while its main drawback is the increase in computational complexity that comes with the use of multiple particles to describe the cell.

A well-known shortcoming of agent-based modelling is the risk to introduce disputable artefacts in the simulations. Within the scope of this work, we have shown that our model adhered well to biology by successfully simulating tissue-level morphological changes based solely on changes triggered at the cellular level, in a bottom-up, emergent fashion. We did this in particular for epithelial bending through apical constriction [42], rosette formation via polarised apical constriction [43], and repulsion-driven lumenogenesis [4, 7]. Nonetheless, some nuance should be added to certain quantitative features of the simulations. For instance, although it is a biological fact that the epiblast lumen’s volume increases as a result of cells drifting apart, the rate of this growth as exhibited in the graph of Fig 3E may not reflect the actual rate curve in mouse embryos. The same could be said of the rate at which the epiblast reshapes (Fig 4K and 4L), or the trophectoderm-induced epiblast velocity in its motion towards maternal sites (Fig 5B). While not invalidating our main conclusions, these quantitative outputs are essentially contingent upon the choice of the potential function (here the Morse potential) and parameter values. This limitation could be overcome by experimenting with other potential functions, searching parameters space, and comparing results against real biological data.

Another weakness of computational modelling is its inability to integrate all possible details of a real-world problem, as this would inevitably increase complexity and demand unavailable computing power. Clearly, efficiency in our simulations was achieved by stripping the model of noticeable features of biological development. One important approximation is that we ignored the hypothetical impact of proliferation, although it is a pervasive phenomenon in both tissues. However, while it may be argued that proliferation plays a non-trivial role in the elongation of trophectodermal cells [10], it is difficult to make a case that proliferation would be central in reshaping the epiblast, or the invagination of the trophectoderm, as previous research has shown that proliferation was not required for epithelial invagination [44]. In fact, this particular lack in our approach could even be considered an advantage, since neglecting proliferation also allowed isolating, hence highlighting the effects of pure mechanical interactions within and between the trophectoderm and the epiblast. Another simplification is that we neglected stochastic effects related for example to cell movements during these embryogenesis episodes. However, in epithelial settings, stochastic effects are often compensated by strong interactions between cells [21]. Furthermore, in general, deterministic models, still exhibit good predictive power while remaining computationally practical [45].

In summary, although relatively abstract and schematic, our computational model and simulations offer new insights into mouse embryo implantation. Looking forward, refinements could combine the effects of mechanical interactions with proliferation and the stochasticity of biological processes to further investigate tissue shape changes. In this way, the variables and parameters in these simulations could be tuned to fit quantitative metrics based on real measurements gathered from implanting embryos.

## Supporting information

S1 Fig Epiblast and trophectoderm population reconstruction. **A**. The rosette-shaped EPI tissue is built by submitting polarised cells in a double epithelial layer to apical constriction. Green arrows indicate the apical surface of the cells, where constriction occurs. **B**. The initial cell population (TE and EPI) is built by adding an epithelial layer to the forming the EPI.
(TIF)

S2 Fig Top view of trophectoderm morphogenesis. **A**. Initial stage with cuboidal cells. **B**. Columnar TE initiating apical constriction. Red arrows highlight cells undergoing apical constriction. In this case, only cells in the middle constrict (light blue) to enable invagination. **C**. Folded TE. **D**. Folded TE after separation from the EPI.
(TIF)

S3 Fig Trophectoderm fosters epiblast movement towards maternal sites (Without lumenogenesis in epiblast). **A**. Snapshots of the simulation of TE and EPI morphogenesis during mouse implantation, and their influence on EPI positioning, taken respectively at *t* = 0 and 6000. **B**. Plot of the pushing distance, which increases with time. **C**. Plot of the elastic energy *E*. Discontinuities mark the start of new morphological events (*t* = 0 and 3000). **D**. Plot of the pushing distance on the epiblast Centre of Mass (CoM), which also increases with time. **E**. Plot of the pushing distance on the cell population Centre of Mass (CoM), which also increases with time. Values of the equation parameters: *J*_EPI_ = *J*_TE_ = 2.5, λ_med_ = λ_χ_ = 2, *ρ* = 1.
(TIF)

S4 FigSensitivity analysis (Supplementary).**A**. Stress-Strain curve (*black*) for a single epithelial cell (34 vertices) with *J* = 2.5 and λ_med_ = λ_χ_ = 2. (*blue*) Linear approximation of the Stress-Strain curve. The elastic modulus of the cell is determined by the slope of this line (*Y* = 2.92). **B**. Plot of the Elastic (Young) modulus of cells as a function of the parameter ratio ($\frac{\lambda_{\chi }}{\lambda_{\text{med}}}$). Young’s modulus is defined and constant for values of $\frac{\lambda_{\chi }}{\lambda_{\text{med}}}$ greater or equal to approximately 0.161. Below this value, simulated cells do not behave as physical materials, and the elasticity modulus cannot be defined as illustrated in the next plot. **C**. Stress-Strain curve (*black*) for a single epithelial cell (34 vertices) with *J* = 2.5, λ_med_ = 2 and λ_χ_ = 0.25. The discontinuity in the curve shows that the set of parameters is not suitable for a cell. **D**. 3D rendering of an epithelial cell with square basis and 42 vertices. **E**. Stress-Strain curve (*black*) for a single epithelial cell (42 vertices) with *J* = 2.5 and λ_med_ = λ_χ_ = 2. (*blue*) Linear approximation of the Stress-Strain curve. The elastic modulus of the cell is determined by the slope of this line (*Y* = 2.75). **F**. Plot of the Elastic (Young) modulus of a cell (42 vertices) as a function of the parameter *J*, the interaction strength between subcellular particles. In order for such a cell (42 vertices) to have equivalent stiffness with the previous type of cell (34 vertices, *J*_34_ = 2.5, *Y*_34_ = 2.92), the parameter *J*_42_ needs to be set to approximately 2.6 (*Y*_42_ = 2.90).(TIF)

S1 Video Simulated morphogenesis during mouse implantation. Trophectoderm cells elongate and then undergo apical constriction, leading the tissue to fold. At the same time, the epiblast remodels from a nearly spherical tissue to a cup-shaped tissue, while also undergoing lumenogenesis.
(MP4)

S2 Video Trophectoderm regulates epiblast shape. Trophectoderm and epiblast undergo their normal development sequences (signle cells elongation followed by invagination of trophectoderm, and reshaping and lumenogenesis in the epiblast). After the trophetoderm is detached from the epiblast, the epiblast bounces back to its nearly spherical shape. This shows that the epiblast broke symmetry and remodelled in the first place under mechanical stress imposed by trophectoderm morphogenesis.
(MP4)
