# Supplementary figures and images for: Computational modelling unveils how epiblast remodelling and positioning rely on trophectoderm morphogenesis during mouse implantation
Source: PLoS One. 2021 Jul 28;16(7):e0254763. doi: 10.1371/journal.pone.0254763 (PMC8318228; doi:10.1371/journal.pone.0254763)

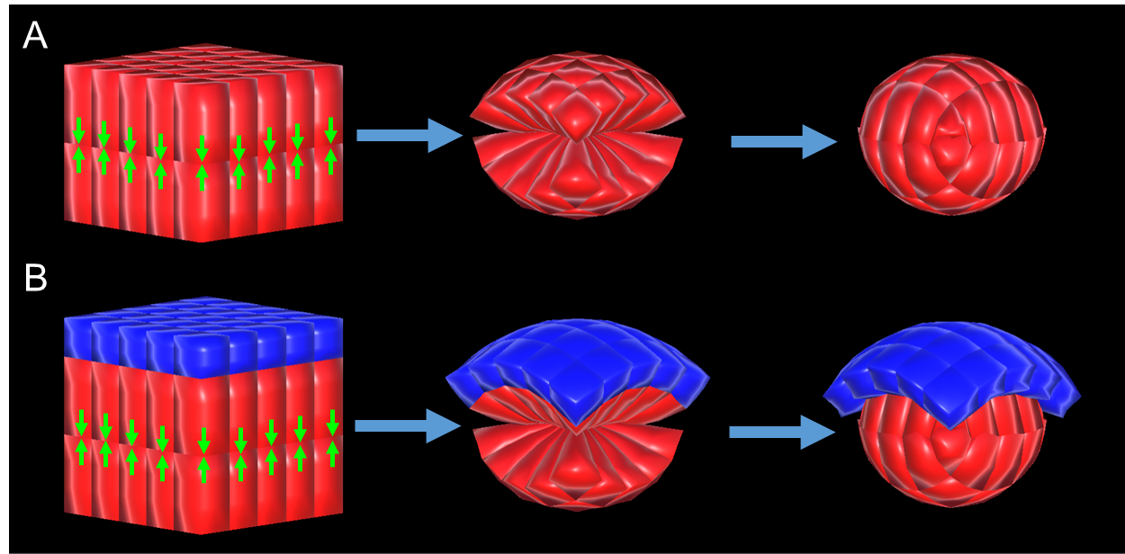

Supplement: S1 Fig — A. The rosette-shaped EPI tissue is built by submitting polarised cells in a double epithelial layer to apical constriction. Green arrows indicate the apical surface of the cells, where constriction occurs. B. The initial cell population (TE and EPI) is built by adding an epithelial layer to the forming the EPI. (TIF) [file pone.0254763.s001.tif]

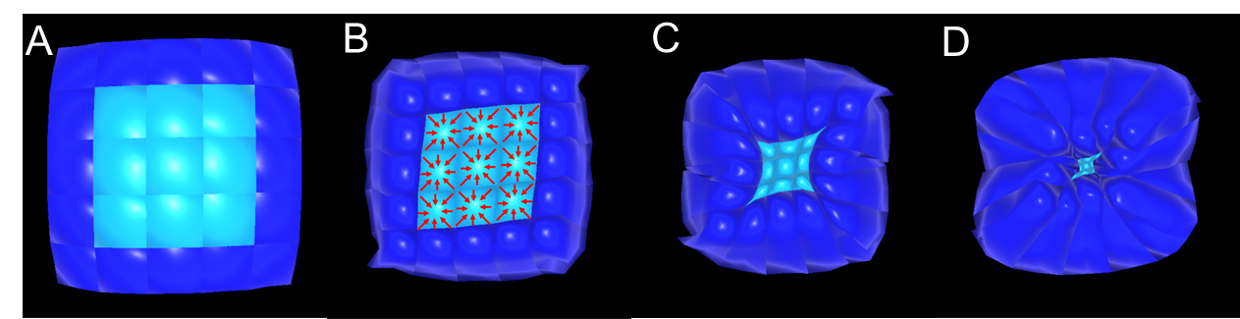

Supplement: S2 Fig — A. Initial stage with cuboidal cells. B. Columnar TE initiating apical constriction. Red arrows highlight cells undergoing apical constriction. In this case, only cells in the middle constrict (light blue) to enable invagination. C. Folded TE. D. Folded TE after separation from the EPI. (TIF) [file pone.0254763.s002.tif]

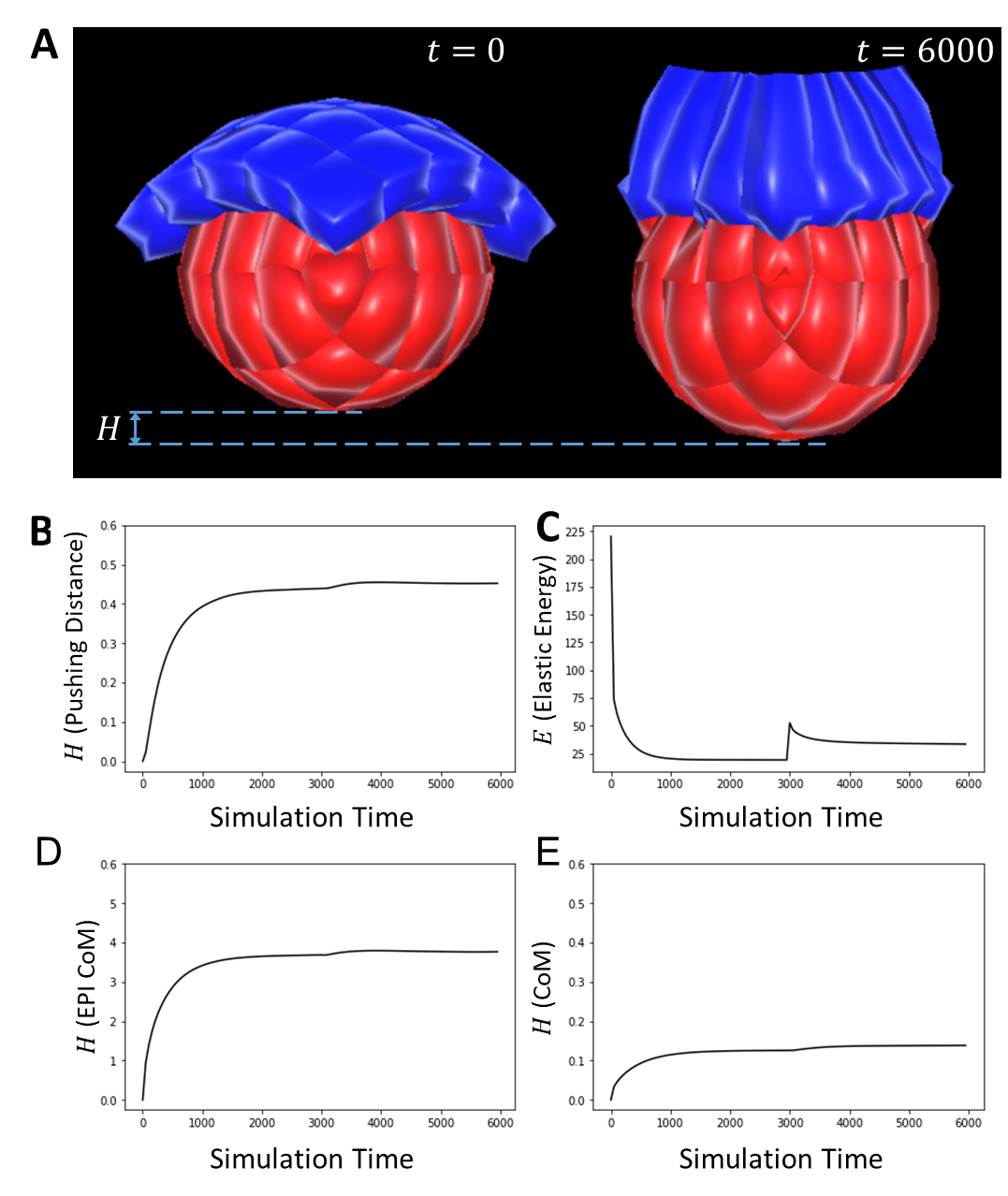

Supplement: S3 Fig — A. Snapshots of the simulation of TE and EPI morphogenesis during mouse implantation, and their influence on EPI positioning, taken respectively at t = 0 and 6000. B. Plot of the pushing distance, which increases with time. C. Plot of the elastic energy E. Discontinuities mark the start of new morphological events (t = 0 and 3000). D. Plot of the pushing distance on the epiblast Centre of Mass (CoM), which also increases with time. E. Plot of the pushing distance on the cell population Centre of Mass (CoM), which also increases with time. Values of the equation parameters: JEPI = JTE = 2.5, λmed = λχ = 2, ρ = 1. (TIF) [file pone.0254763.s003.tif]

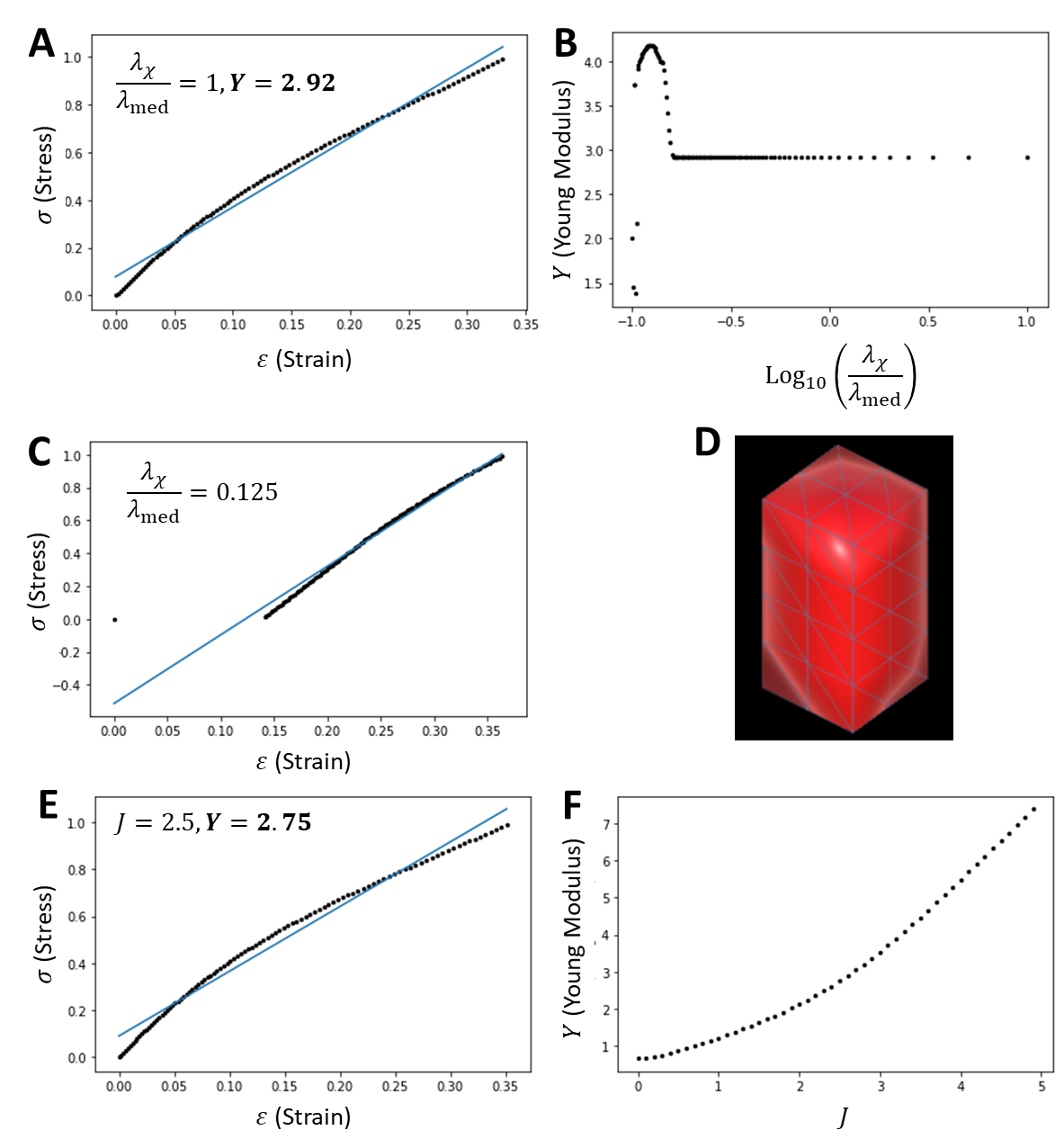

Supplement: S4 Fig — A. Stress-Strain curve (black) for a single epithelial cell (34 vertices) with J = 2.5 and λmed = λχ = 2. (blue) Linear approximation of the Stress-Strain curve. The elastic modulus of the cell is determined by the slope of this line (Y = 2.92). B. Plot of the Elastic (Young) modulus of cells as a function of the parameter ratio (λχλmed). Young’s modulus is defined and constant for values of λχλmed greater or equal to approximately 0.161. Below this value, simulated cells do not behave as physical materials, and the elasticity modulus cannot be defined as illustrated in the next plot. C. Stress-Strain curve (black) for a single epithelial cell (34 vertices) with J = 2.5, λmed = 2 and λχ = 0.25. The discontinuity in the curve shows that the set of parameters is not suitable for a cell. D. 3D rendering of an epithelial cell with square basis and 42 vertices. E. Stress-Strain curve (black) for a single epithelial cell (42 vertices) with J = 2.5 and λmed = λχ = 2. (blue) Linear approximation of the Stress-Strain curve. The elastic modulus of the cell is determined by the slope of this line (Y = 2.75). F. Plot of the Elastic (Young) modulus of a cell (42 vertices) as a function of the parameter J, the interaction strength between subcellular particles. In order for such a cell (42 vertices) to have equivalent stiffness with the previous type of cell (34 vertices, J34 = 2.5, Y34 = 2.92), the parameter J42 needs to be set to approximately 2.6 (Y42 = 2.90). (TIF) [file pone.0254763.s004.tif]
